# Neuregulin, an Effector on Mitochondria Metabolism That Preserves Insulin Sensitivity

**DOI:** 10.3389/fphys.2020.00696

**Published:** 2020-06-23

**Authors:** Anna Gumà, Francisco Díaz-Sáez, Marta Camps, Antonio Zorzano

**Affiliations:** ^1^Department of Biochemistry and Molecular Biomedicine, Faculty of Biology, University of Barcelona, Barcelona, Spain; ^2^CIBER de Diabetes y Enfermedades Metabólicas Asociadas (CIBERDEM), Instituto de Salud Carlos III, Madrid, Spain; ^3^Institute of Biomedicine of the University of Barcelona (IBUB), University of Barcelona, Barcelona, Spain; ^4^Institute for Research in Biomedicine (IRB Barcelona), The Barcelona Institute of Science and Technology, Barcelona, Spain

**Keywords:** mitochondria, neuregulin, ErbB receptors, obesity, diabetes, insulin resistance

## Abstract

Various external factors modulate the metabolic efficiency of mitochondria. This review focuses on the impact of the growth factor neuregulin and its ErbB receptors on mitochondria and their relationship with several physiopathological alterations. Neuregulin is involved in the differentiation of heart, skeletal muscle, and the neuronal system, among others; and its deficiency is deleterious for the health. Information gathered over the last two decades suggests that neuregulin plays a key role in regulating the mitochondrial oxidative machinery, which sustains cell survival and insulin sensitivity.

## Neuregulin, a Members of the Epidermal Growth Factor Family

The early 1990s saw the publication of several articles searching for the ligand of a relevant protooncogene, *c-neu*, also known as ErbB2 receptor (erythroblastic leukemia viral oncogene homolog 2 receptor) or HER2 in humans, associated with malignancy and poor prognosis in breast, ovarian, gastric, and endometrial cancers. The authors of these reports named the hypothetical ligand for the ErbB2 receptor, a 44–45 kD glycoprotein, in distinct ways, including heregulin (HRG) (Holmes et al., 1992) and neu differentiation factor (NDF) (Peles et al., 1992). Several laboratories cloned and identified other members of the family, and the common term neuregulin (NRG) was proposed encompassing them all (Marchionni et al., 1993; Fischbach and Rosen, 1997).

NRG belongs to the epidermal growth factor (EGF) family since it contains the domain that characterizes all the members of this family, the EGF-like domain, which allows binding to the ErbB tyrosine kinase receptor family (Carraway and Burden, 1995; Gumà et al., 2010). NRG subfamily members are encoded by various genes, the products of *nrg-1* to *4* genes being the most widely studied. Most of these subfamily members contain a transmembrane domain. The bioactive *EGF-like* domain is located extracellularly, in the N terminus portion, and it can be released upon proteolysis by metalloproteases (Montero et al., 2000; Ozaki et al., 2004). Such proteases target specific sites at the NRG juxtamembrane extracellular region. Upon release, the *EGF-like* domain of NRG binds to ErbB receptors. In contrast to what was expected, NRG does not bind directly to ErbB2 receptor (Peles et al., 1993), but to ErbB3 and ErbB4 (Plowman et al., 1993b; Carraway and Cantley, 1994; Tzahar et al., 1994). NRG binding to ErbB3 or ErbB4 triggers preferential heterodimerization with the orphan receptor ErbB2 or, in its absence, with ErbB1 (also known as the EGF receptor, EGFR) (Carraway et al., 1994; Alimandi et al., 1995; Graus-Porta et al., 1995; Riese et al., 1995; Pinkas-Kramarski et al., 1996). ErbB3 is a kinase-death receptor, whereas ErbB4 displays both binding and kinase activity, the latter having a wider spectrum of NRG ligands (Jones et al., 1999).

NRG is released by cells of endothelial, mesenchymal, and neuronal origin, while ErbB receptors are located close to the ligand, generating local autocrine, paracrine, or even juxtacrine actions (Gumà et al., 2010). More recently, a member of the NRG subfamily, NRG-4, has emerged as an endocrine factor, which is addressed later.

## Role of Neuregulin and ErbB Receptors on Cell Survival and Oxidative Stress

Anthracyclines such as doxorubicin are widely used as chemotherapeutic agents for the treatment of cancer. These drugs induce cardiomyopathy, and there is evidence that disturbances at the NRG/ErbB axis play a crucial role in the development of anthracycline-dependent cardiotoxicity (Ghigo et al., 2016). In cancers that overexpress the product of the oncogene *erbB2*, therapy combines the use of anthracyclines with ErbB2-blocking antibodies (trastuzumab or herceptin). In such cases, the cardiotoxic effect of anthracyclines is enhanced by ErbB2 blockage (Slamon et al., 2001; Keefe, 2002). There is evidence indicating that NRG-1, acting on ErbB4/ErbB2 receptors, plays a critical role in heart development. In this regard, knockout mice for NRG-1 (Meyer and Birchmeier, 1995), ErbB4 (Gassmann et al., 1995), and ErbB2 (Lee et al., 1995) die at mid-embryo life due to altered heart ventricular trabeculation. The lethality observed in knockout mice led to the development of new approaches to analyze the relevance of NRG and ErbB receptors in adult heart. Mice with a postnatal conditional ErbB2 mutation in ventricular cardiomyocytes show a severe dilated cardiomyopathy at the second month of age, indicating that ErbB2 has a cardioprotective role in adulthood (Ozcelik et al., 2002). Another strategy was based on the use of heterozygous NRG-1 knockout mice, which show accelerated systolic failure and higher mortality in response to doxorubicin (Liu et al., 2005). The treatment with a bioactive recombinant NRG-1β counteracts doxorubicin-dependent reduction of cardiomyocyte contractility and myofilament disarray in cardiomyocytes (Sawyer et al., 2002; Timolati et al., 2006). In this regard, NRG-1 promotes cell survival via a phosphatidylinositol 3-kinase-dependent mechanism that requires the activity of ErbB4/ErbB2 receptors (Fukazawa et al., 2003; Bian et al., 2009). Overexpression of ErbB2 in heart reduces mitochondrial ROS production in response to doxorubicin (Belmonte et al., 2015). In contrast, primary cultures of neonatal rat ventricular myocytes exposed to anti-ErbB2 antibody for 24 h show impaired mitochondrial function and cellular energy (Grazette et al., 2004). A single challenge of NRG-1β triggers cellular reprogramming in cardiomyocytes not only by improving the mitochondrial oxidative capacity and the defense against oxidative stress, thereby enhancing cell survival, but also by increasing the protein synthesis and the glycolytic metabolism that contribute to cardiomyocyte hypertrophy. These findings highlight the protective function of NRG in the heart (Giraud et al., 2005). The observation that NRG prevents the deleterious effects of oxidative stress is supported by previous reports indicating that the release of this growth factor by cardiac endothelial cells prevents cardiomyocyte apoptosis by regulating ROS levels, in a manner that involves ErbB4 activation (Kuramochi et al., 2004). This protective role of NRG against oxidative stress has also been reported in other cell types such as the neuronal PC12 cells (Goldshmit et al., 2001).

The beneficial effect of ErbB2 in differentiated tissues contrasts with the consequences of its overexpression, which is associated with cancer. In this context, the overexpression of ErbB2 also targets mitochondria, although acting in such a manner that contributes to cell transformation. Regarding ErbB2 action on mitochondria proteins, two studies have provided valuable insight. On the one hand, ErbB2 antagonizes apoptosis in cancer cells by physically interacting with the mitochondrial protein p53 upregulated modulator of apoptosis (PUMA), a potent apoptosis inducer. ErbB2 signals the phosphorylation of PUMA in tyrosine residues promoting its degradation by the proteasome (Carpenter et al., 2013). In addition, ErbB2 tyrosine kinase signaling induces the phosphorylation of the mitochondrial creatine kinase 1 (MtCK1, located at the intermembrane space) on tyrosine 153 (Y153) in breast cancer cells. Y153 phosphorylation stabilizes MtCK1 protein, thereby increasing the phosphocreatine energy shuttle and promoting proliferation (Kurmi et al., 2018). Moreover, studies in cancer cells and patient samples show that when ErbB2 is overexpressed, it translocates from the plasma membrane to mitochondria. The increase of ErbB2 in mitochondria negatively regulates mitochondrial respiration, membrane potential, and ATP synthesis while enhancing anaerobic glycolysis, thereby increasing lactate production (Ding et al., 2012). Furthermore, ErbB2 induces the overexpression of the uncoupling protein 2 (UCP2) in cancer cells. In normal conditions, UCP2 is associated with glucose tolerance and insulin sensitivity, as it controls the production of reactive oxygen species (ROS) during respiration (Dalgaard, 2011, 2012). In contrast, the UCP2 overexpression leads to the uncoupling of mitochondria, which contributes to enhance anaerobic glycolysis in cancer cells (Patel et al., 2013).

## Involvement of Neuregulin and ErbB Receptors in Adaptive Changes to Oxidative Metabolism

The generation of genetically modified mouse models for NRG-1 and for ErbB receptors has allowed the observation of serious alterations in the development of the central and peripheral nervous system, beyond the effects on heart development already described (Lee et al., 1995; Meyer and Birchmeier, 1995; Carraway, 1996). In response to synaptic activity, NRG-1 is released by neuronal axons and binds to ErbB receptors which are concentrated at the neuronal and neuromuscular post-synaptic plates as well as at central and peripheral glial cells (Altiok et al., 1995; Burden and Yarden, 1997; Buonanno and Fischbach, 2001), inducing both glia maturation and postsynaptic plate formation (Falls et al., 1993; Marchionni et al., 1993; Chen et al., 1994; Corfas et al., 1995; Dong et al., 1995; Goodearl et al., 1995; Jo et al., 1995; Morrissey et al., 1995; Woldeyesus et al., 1999; Wolpowitz et al., 2000; Yang et al., 2001). Synaptic activity causes muscle contraction, and there are multiple evidences indicating that NRG-1 participates in contraction events that modulate muscle metabolism. First, muscle also expresses NRG-1, and it is essential for the myogenesis (Kim et al., 1999; Suarez et al., 2001), acting in an additive manner with the insulin-like growth factor I, IGF-I (Florini et al., 1996). Second, muscle fibers release NRG-1 in response to muscle contraction (Lebrasseur et al., 2003; Canto et al., 2006), and the impairment of NRG-1 action during an acute stimulus of contraction leads to a decrease in glycogen, ATP, and phosphocreatine muscle content (Canto et al., 2006). In this sense, it is well known that muscle contraction has rapid effects inducing glucose uptake in order to sustain energy reservoirs. Contraction effects on glucose uptake take place in an additive manner to the insulin action, by translocation of GLUT4 glucose transporters to surface membranes (Ploug et al., 1998). Interestingly, insulin and NRG-1 also have additive effects inducing glucose transport and the translocation of glucose transporters to surface membranes in muscle cells (Suarez et al., 2001; Canto et al., 2004). Third, NRG-1 has long-term effects on muscle cells that resemble those of muscle adaptation to exercise. Chronic effects of exercise result in the increase in mitochondrial biogenesis (Takahashi and Hood, 1993; Hood, 2001) and mitochondrial respiratory activity, thus enhancing the capacity to oxidize carbohydrates and fatty acids (Holloszy, 1967; Mole et al., 1971). This is relevant since insulin resistance has been associated with impaired mitochondrial activity (Mootha et al., 2003; Patti et al., 2003; Petersen et al., 2003, 2004; Lowell and Shulman, 2005), and exercise is indicated as a therapeutic intervention to prevent and treat insulin resistance (Baar et al., 2002; Hawley, 2004). In this sense, long-term treatment of muscle cells with the bioactive recombinant NRG-1 isoform, heregulin-β1 177–244 (Hrg), induces glucose and fatty acid oxidation through an increase in total mitochondria content and membrane potential as well as inducing the expression of mitochondrial respiratory chain complexes, resulting in improved insulin sensitivity (Canto et al., 2007). These effects are a consequence of the NRG-1 action inducing the expression of the transcription factor, peroxisome proliferator–activated receptor β/δ (PPARβ/δ), and the PPARγ coactivator-1α (PGC-1α) (Canto et al., 2007), both involved in mitochondrial biogenesis and activity (Wu et al., 1999). Current data indicate that PGC-1α induces the expression of mitofusin 2 (MFN2) gene (Soriano et al., 2006), an outer mitochondrial membrane protein that stimulates mitochondrial fusion, mitochondrial oxidative activity, and, in turn, insulin sensitivity (Bach et al., 2003; Sebastian et al., 2012). It would be pertinent to study whether NRG-1 regulates MFN2 expression and, consequently, whether it can modulate mitochondrial dynamics in muscle. Recent evidences support this view. In neonatal cardiomyocytes, submitted to hypoxia/reoxygenation injury, Hrg contributes to cardiomyocytes survival by restoring the expression of MFN1 and MFN2 (Zhang et al., 2020). All together suggest that NRG-1 has a prevalent role in muscle adaptation to an oxidative metabolism.

## Neuregulin 4, a New Adipokine That Enhances Browning in Adipose Tissue

In 2014, two groups reported that NRG-4 is highly expressed and released by adipose tissues, especially by brown adipose tissue (BAT), thus being considered an adipokine (Rosell et al., 2014; Wang et al., 2014). Despite this, some other tissues such as the liver, lung, and pancreas are capable of expressing NRG-4, although in much lower amounts. NRG-4 exerts a plethora of effects that ultimately regulate energy metabolism and insulin sensitivity. Despite the abundance of NRG-4 in BAT, this protein is dispensable for defense against cold exposure-induced hypothermia in NRG-4 null mice (Wang et al., 2014). However, NRG-4 expression is upregulated in white adipose tissue (WAT) upon cold exposure (Rosell et al., 2014). In this condition, NRG-4 increases innervation by promoting neurite growth and contributes to the transition from white to beige adipocytes. Beige adipocytes can be differentiated from white adipocytes by their increased thermogenic capacity. Beige adipocytes induce uncoupling protein 1 (UCP1) expression upon adrenergic stimulus and increase mitochondrial oxygen consumption, thereby allowing a better control of body energy balance and insulin sensitivity (Wu et al., 2012). Hence, NRG-4 might be a key factor for the acquisition of BAT features in WAT depots. In this regard, there is a positive correlation between the expression of NRG-4 and beige markers such as the UCP1, UCP3, and Transmembrane Protein 26 (TMEM26) in human WAT (Comas et al., 2019). Studies in 3T3-L1 adipocytes show that the administration of NRG-4 inhibits lipogenesis and promotes the expression of markers of beige cells such as proton/amino acid transporter 2 (PAT2) and cluster of differentiation 137 (CD137), as well as markers of browning. such as UCP1 and PR domain zinc finger protein 16 (PRDM16) (Zeng et al., 2018).

Genetically modified mouse models in which NRG-4 expression is blocked or enhanced have been generated with the aim to study the effect of this protein on adipose tissue. Initially, it was reported that NRG-4 null mice did not show alterations in insulin sensitivity when treated with a control diet. However, upon treatment with a high-fat diet (HFD), these mice became more obese and showed increased plasma triacylglycerol levels and higher fasting blood glucose and insulin levels (Wang et al., 2014). In this regard, NRG-4 expression is reduced in WAT in obesity, both in mouse models and humans (Wang et al., 2014), and adiposity negatively correlates with NRG-4 expression in WAT, but not in BAT (Chen et al., 2017). Moreover, studies comparing a cohort of body mass index-matched individuals reveals that NRG-4 mRNA levels in WAT are lower in those individuals with impaired glucose tolerance or type 2 diabetes than in those with normal glucose tolerance (Wang et al., 2014). In contrast, NRG-4 transgenic mice show enhanced whole body glucose metabolism and, consequently, reduced obesity and insulin resistance compared to control animals (Wang et al., 2014; Chen et al., 2017). Mice with adipose-selective overexpression of NRG-4, treated with a HFD, show greater oxygen consumption and energy expenditure than control mice (Chen et al., 2017). In the same study, microarray gene expression analysis of WAT from wild-type (WT) and NRG-4 transgenic mice revealed the upregulation of a cluster of genes related to mitochondrial function and energy expenditure (Chen et al., 2017).

Interestingly, NRG-4 overexpression promotes a healthier adipokine profile during obesity. Hence, NRG-4 and adiponectin expression change in a similar manner, while NRG-4 overexpression counteracts the expression of proinflammatory cytokines involved in obesity-induced adipose tissue inflammation, such as tumor necrosis factor α (TNFα) and interleukin 1β (IL1β) (Wang et al., 2014; Chen et al., 2017). In turn, the expression of NRG-4 is inhibited by treatment with the inflammatory cytokine TNFα in 3T3-L1 adipocytes (Wang et al., 2014; Chen et al., 2017) and its expression is recovered by inhibition of NF-κB or activation of PPARγ in these cells (Chen et al., 2017). At the same time, the expression of the pro-inflammatory cytokines IL1β and TNFα is upregulated in NRG-4-deficient adipose tissue in mice (Wang et al., 2014; Chen et al., 2017). Therefore, the reduction in NRG-4 expression that is observed in obesity could be a consequence of the low-grade chronic inflammatory signaling present in WAT.

More recently, the role of NRG-4 in adipose tissue vascularization was explored since the pathological adipose tissue expansion that occurs in obesity is often accompanied by a reduction in blood vessels, thereby leading to hypoxia (Corvera and Gealekman, 2014). Hypoxia exacerbates the manifestation of an inflammatory phenotype in adipocytes (Pasarica et al., 2009, 2010). NRG-4 triggers endothelial angiogenic functions and angiogenesis both *in vitro* and *in vivo* (Nugroho et al., 2018a). NRG-4 -/- mice show a reduction in blood vessels in both BAT and WAT, but unlike the aforementioned studies (Wang et al., 2014; Chen et al., 2017) in the work of Nugroho et al., NRG-4 -/- mice increase in body weight and adiposity even under a normal diet, without altering food intake when compared with WT mice. Moreover, NRG-4 -/- mice showed reduced adiponectin expression in WAT, reduced insulin sensitivity, impaired glucose tolerance, and a decrease in oxygen consumption without a decline in physical activity (Nugroho et al., 2018a). In contrast, transgenic mice overexpressing NRG-4 in adipocytes, under the control of the promoter aP2, and treated with a HFD, showed enhanced expression of vascular endothelial growth factor (VEGF) (Chen et al., 2017) which is involved in the growth of blood vessels and increased blood vessel density (Nugroho et al., 2018b). As previously described, NRG-4 transgenic mice subjected to a HFD show a decrease in the expression of inflammatory markers such as IL1β, IL6, and TNFα in WAT. In addition, transgenic mice have a higher insulin sensitivity and glucose tolerance than WT mice (Nugroho et al., 2018b). Recent data indicate that NRG-1 is a hypoxia-inducible factor 1α (HIF1α) suppressor in neurons (Yoo et al., 2019). Since adipose tissue hypoxia is one of the first physiopathological changes in WAT in obesity and leads to HIF1α and nuclear factor-kappa B (NF-κB) activation (Sun et al., 2011), the role of NRG-4 in inducing vascularization, thereby preventing hypoxia, contributes to the maintenance of a healthy metabolic profile and absence of inflammation.

## Neuregulin-4 Targets ErbB4 Receptor

NRG-4 specifically binds to ErbB4 receptor (Harari et al., 1999). ErbB4 is highly expressed in the central nervous system (Plowman et al., 1993a, b; Zhang et al., 1997) and also in muscle, heart, pancreas, salivary gland, and lung (Plowman et al., 1993a; Gassmann et al., 1995; Pinkas-Kramarski et al., 1997). Interestingly, ErbB4 is one of the genes linked to obesity and diabetes, as shown by studies of various International Consortiums such as the ADIPOGen and GENIE Consortiums. ErbB4 locates in caveolar microdomains in cardiomyocytes (Zhao et al., 1999). Upon ligand binding, ErbB4 rapidly leaves this site in what is considered a mechanism of receptor desensitization in the continuous presence of the ligand (Zhao et al., 1999). Besides, it has been shown that, after stimulation with NRG-1, ErbB4 is recruited to the lipid raft fraction of neuronal cell membranes. This recruitment plays a critical role in NRG signaling and in the modulation of synaptic plasticity in the brain (Ma et al., 2003). Caveolin-1 is an essential protein component of caveolae but cellular organelles such as mitochondria, nuclei, and endoplasmic reticuli are also rich in caveolins. Caveolin-1 knockout mice have cholesterol-dependent mitochondrial dysfunction and susceptibility to apoptosis (Bosch et al., 2011). Caravia et al. (2015) reviewed the relation between caveolin-1 and mitochondria and suggested that this protein acts as a “warning sign” for mitochondria. Adipocytes are rich in caveolae, and the presence of ErbB4 in caveolin-rich membranes suggests that NRG-4 signaling on mitochondrial metabolism is initiated in caveolae. Although ErbB4 expression decreases during adipogenesis (Zeng et al., 2018), the relevance of ErbB4 has been examined in adipose tissue as NRG-4 has been observed to protect adipocytes against the effects of a HFD (Zeng et al., 2018). Since ErbB4 null mice die at mid-embryo life due to impaired heart development, a heart-rescued ErbB4 deletion mouse model was generated to analyze ErbB4 involvement in the protective effect of NRG-4 against a medium-fat diet (Zeng et al., 2018). Mice lacking ErbB4 developed obesity, dyslipidemia, hepatic steatosis, hyperglycemia, hyperinsulinemia, and insulin resistance.

Severe inflammation and M1 macrophage polarization occur in both inguinal and epididymal WAT of mice with ErbB4 deletion (Zeng et al., 2018). Previous evidence of the anti-inflammatory role of the NRG-4/ErbB4 axis came from studies on inflammatory bowel diseases, such as Crohn’s disease and ulcerative colitis (Frey et al., 2009; Bernard et al., 2012; McElroy et al., 2014; Schumacher et al., 2017). NRG-4 expression is suppressed in inflammatory bowel disease (Bernard et al., 2012), whereas exogenous treatment with this protein blocks enterocyte apoptosis in rodent models of intestinal inflammation and is therefore protective (Bernard et al., 2012; McElroy et al., 2014). In this regard, *in vivo* analysis of macrophages from a dextran sulfate sodium (DSS)-induced colitis model in C57Bl/6 mice revealed a dramatic reduction in NRG-4 expression due to the underlying colonic inflammation. Exogenous treatment with NRG-4 ameliorated this inflammation, hence reducing the expression of pro-inflammatory cytokines. In contrast, ErbB4 is selectively overexpressed by pro-inflammatory macrophages during inflammation, and when this receptor is activated by NRG-4, it promotes M1 macrophages death (Schumacher et al., 2017). This deleterious effect on macrophages involves ErbB4 cleavage upon NRG-4 binding and intracellular fragment translocation to mitochondria, which reduces mitochondrial membrane potential and triggers apoptosis (Schumacher et al., 2017).

These lines of evidence point to the potential of NRG-4 as an efficient promoter of pro-inflammatory macrophage clearance. This function is critical to prevent chronic inflammation since defects in such clearance can lead to auto-inflammatory diseases and may contribute to metabolic syndrome. In all, NRG-4 secreted by WAT may play a key role in preventing inflammation, and the decrease in its expression observed in obesity may exacerbate inflammation driven by infiltrated macrophages.

## Neuregulin as an Endocrine Factor That Protects Against Insulin Resistance

Neuregulins were classically considered as local growth factors. However, NRG-1 has been detected in plasma, and its presence has been associated with heart, lung, and neuronal diseases, either as a consequence of the release of NRG-1 derived of the tissue damage or due to the overexpression of such protective factors. Therefore, NRG-1 in plasma has been examined as a possible diagnostic marker for various diseases that can alter tissue integrity and thus compromise cell survival. Plasma NRG-1 has been linked to the severity of chronic heart failure (Ky et al., 2009) and coronary artery disease (Geisberg et al., 2011) as well as acute lung injury, correlating with inflammation (Finigan et al., 2013). Regarding neuronal diseases, NRG-1 is found in the plasma of Parkinson’s disease patients and its plasma levels are associated with those of the cerebral spinal fluid (Hama et al., 2015). NRG-1 is also detected in the plasma of Alzheimer’s disease patients (Chang et al., 2016) and it has been proposed as a marker of the onset of this condition.

New evidence supports the notion that the adipokine NRG-4 has an endocrine role, exerting metabolic effects locally in adipose tissues, as mentioned above, but also acting on distal tissues with special incidence on the liver (Wang et al., 2014; Ma et al., 2016). Studies using NRG-4 null mice as well as adipose tissue-overexpressing NRG-4 mouse models demonstrated that NRG-4 protects against HFD-induced insulin resistance by attenuating hepatic *de novo* lipogenesis (Wang et al., 2014; Ma et al., 2016) and activating mitochondrial fatty acid oxidation and ketogenesis (Chen et al., 2017). Others confirmed these results using a hydrodynamic gene transfer method to overexpress NRG-4, which targets mainly liver and also adipose tissues, in HFD-treated obese mice (Ma et al., 2016). In that study, overexpression of hepatic NRG-4 ameliorated chronic inflammation, improved insulin resistance, and prevented HFD-induced weight gain and fatty liver (Ma et al., 2016). In another study, NRG-4-overexpressing adipose-derived mesenchymal stem cells were transplanted intravenously into HFD-fed mice (Wang W et al., 2019). NRG-4 overexpression ameliorated insulin resistance by attenuating hepatic steatosis. Hepatic NRG-4 signaling is a checkpoint for the progression of steatosis to nonalcoholic steatohepatitis (NASH). NASH is strongly associated with obesity and metabolic syndrome and characterized by liver inflammation and fibrosis, which can develop into hepatocellular carcinoma (HCC). NRG-4 null mice show accelerated liver injury, fibrosis, inflammation, and cell death in an induced-NASH condition (Guo et al., 2017). Transgenic overexpression of NRG-4 in adipose tissues attenuates hepatocyte death by reducing phosphorylated levels of cJun Kinase (JNK) (Guo et al., 2017), a kinase whose signaling induces inflammation and promotes hepatocyte apoptosis (Seki et al., 2012). These findings highlight the relevance of NRG-4 in sustaining insulin sensitivity by targeting liver metabolism.

ErbB4 expression is higher in skeletal muscle than in liver (Plowman et al., 1993a, b), thereby suggesting potential metabolic effects of NRG in the former. In this regard, the intraperitoneal administration of recombinant NRG, Hrg, before a glucose tolerance test, leads to a rapid increase in glucose utilization in liver, but not in skeletal muscle, and a significant reduction in glycemia both in control and type 2 diabetic rats (Lopez-Soldado et al., 2016). The lack of effects of Hrg may be explained by the difficulty of NRG to access ErbB4 in muscle. In skeletal muscle, ErbB4 locates in the neuromuscular junction (Zhu et al., 1995) and in the invaginations of surface membranes, known as transverse tubules, T-tubules, which are rich in caveolin-3 (Ozcelik et al., 2002; Ueda et al., 2005). Some authors reported that T-tubules open during muscle contraction, otherwise narrowly constraining the arrival of molecules (Wang et al., 1996); however, this is still controversial (Ploug et al., 1998). During contraction, muscle releases NRG-1, which acts locally, through ErbB4, to increase glucose uptake and adapt muscle metabolism to energy requirements (Canto et al., 2006). The addition of external recombinant NRG does not improve the effects of the endogenous secreted NRG in contracting muscle (Canto et al., 2006) or in resting muscle where its effect on glucose uptake is scarce (Suarez et al., 2001). However, the access of circulating NRG to the liver may be favored by this organ, being highly blood irrigated with fenestrated blood vessels, and although hepatocytes express lower levels of ErbB4 than muscle fibers, they may be enough to mediate the constitutive actions of NRG.

With regard to the role of NRG-4 as an endocrine factor, the plasma levels of NRG-4 and the possible association with obesity, type 2 diabetes and cardiovascular diseases-related to metabolic syndrome have been examined in humans (Dai et al., 2015; Cai et al., 2016; Jiang et al., 2016; Kang et al., 2016; Yan et al., 2017, 2018; Tian et al., 2019; Wang R et al., 2019). However, this relationship is still controversial. Plasma NRG-4 has been reported to be decreased in obese children (Wang R et al., 2019) and adults (Cai et al., 2016) and in patients newly diagnosed with type 2 diabetes mellitus (Yan et al., 2017, 2018) while it is inversely associated with subclinical cardiovascular disease in obese adults (Jiang et al., 2016) and with the severity of coronary artery disease (Tian et al., 2019). In contrast, other authors described no changes in serum NRG-4 in adults with NASH disease (Dai et al., 2015) and even increases in newly diagnosed type 2 diabetic patients (Kang et al., 2016). All these studies were done exclusively on samples obtained from Asian populations. Further research covering a wider range of ethnicity and greater number of samples is required to clarify this question.

Given the capacity of NRG to reverse alterations associated with metabolic syndrome and cardiovascular diseases, some studies have addressed the potential of human recombinant NRG-1 as a therapeutic strategy (Gao et al., 2010; Jabbour et al., 2011). In this regard, long-term treatments involving a high dose of this recombinant protein were required due to the short half-life of the protein. In order to stabilize recombinant NRG, a fusion protein containing the EGF-like domain of human NRG-1 and the Fc domain of human IgG1 was generated (Zhang et al., 2018). Intraperitoneal administration of the NRG-1 fusion protein to HFD-induced obese mice led to a lower expression of gluconeogenic enzymes in liver, thereby resulting in lower blood glucose and thus improved insulin sensitivity. Interestingly, the administration of NRG-1 fusion protein to obese mice induced the depolarization of hypothalamic POMC neurons and increased their firing rate, hence stimulating satiety and thus a reduction in food intake (Zhang et al., 2018). The administration of this fusion protein also increased the expression of the hepatokine fibroblast growth factor 21 (FGF21), which is involved in whole body energy homeostasis (Owen et al., 2015; Zhang et al., 2018), although NRG-1 effects were independent of the increase in FGF21 (Zhang et al., 2018).

## Perspectives on the Role of Neuregulin as an Anti-Inflammatory Factor

**FIGURE 1:**
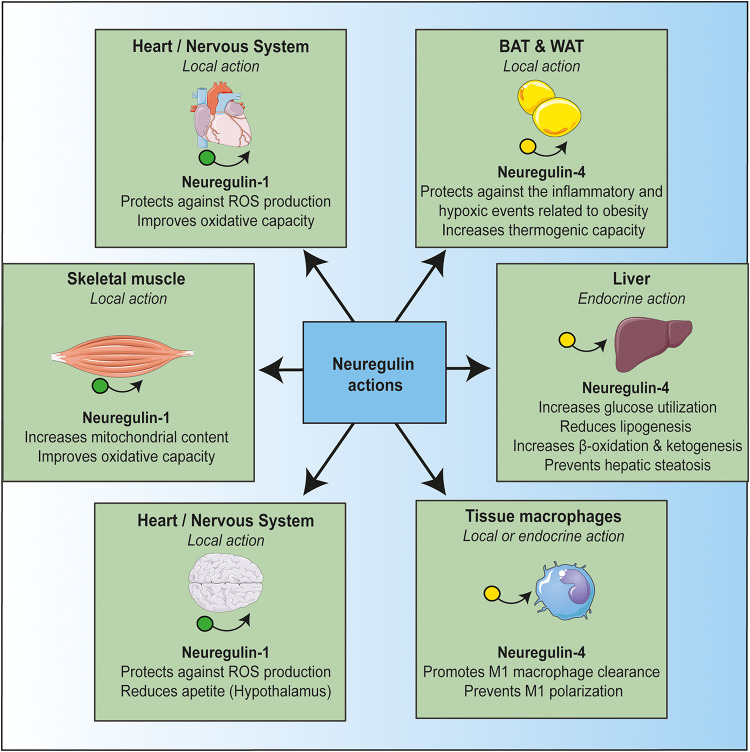
Protective actions of neuregulin on different tissues. Neuregulin, acting locally or as an endocrine factor, protects against metabolic stress and inflammation in a manner that is tightly linked to mitochondria function. Schematic art pieces used in this figure were provided by Servier Medical Art (http://servier.com/Powerpoint-image-bank). Servier Medical Art by Servier is licensed under a Creative Commons Attribution 3.0 Unported License.

The studies reviewed indicate that NRG plays several key roles. It maintains a healthy cellular status in multiple tissues, it safeguards mitochondrial homeostasis, and it prevents alterations driven by inflammation (Figure 1). The role of NRG in mitochondrial activity may be related to its anti-inflammatory effects. Indeed, NRG may regulate metabolic tissue inflammation by modulating mitochondrial function. In this regard, there is increasing evidence that mitochondrial dysfunction is the primary cause of many auto-inflammatory conditions (Dela Cruz and Kang, 2018). Mitochondria are a major source of damage-associated molecular patterns (DAMPS) such as mitochondrial DNA (mtDNA) (Rodriguez-Nuevo et al., 2018) and mitochondrial reactive oxygen species (mROS) (Rimessi et al., 2016). Mitochondria is one of the primary sources of cellular ROS, which is produced at various sites, but especially by complex I and complex III activities (Quinlan et al., 2013). Oxidative stress can initiate inflammation through the NF-κB pathway, thereby increasing the expression of several pro-inflammatory cytokines (Morgan and Liu, 2011). In addition, ROS can activate a leucine-rich repeat-containing protein (NLRP) family member, namely NLRP3, at the inflammasome to promote IL1β cleavage and pyroptosis. In turn, NLRP3 can induce ROS production, thereby further increasing inflammation (Heid et al., 2013). This positive inflammatory feedback loop, caused by oxidative stress, feeds many inflammatory alterations that are involved in the development of metabolic syndrome and type II diabetes. The treatment of inflammation, focusing on mitochondrial homeostasis, emerges as a novel therapeutic field. In this regard, NRG may prevent this inflammatory loop by blocking NF-κB activation and promoting macrophage clearance, thereby improving insulin sensitivity.

In summary, given the protective role of NRG against insults that impair cell energy stability and thus compromise cell survival, this growth factor emerges as a potential drug candidate for the treatment of multiple pathologies, particularly those involving inflammatory and metabolic dysregulation such as obesity and type 2 diabetes. Nowadays, it is ahead of our knowledge, and future studies will have to delineate the conditions in which neuregulin could be useful for therapeutic application.

## Author Contributions

AG organized, wrote, and reviewed the manuscript. FD-S, MC, and AZ wrote and reviewed the manuscript. All authors contributed to the article and approved the submitted version.

## Conflict of Interest

The authors declare that the research was conducted in the absence of any commercial or financial relationships that could be construed as a potential conflict of interest.
